# A Review of Primary Vasculitis Mimickers Based on the Chapel Hill Consensus Classification

**DOI:** 10.1155/2020/8392542

**Published:** 2020-02-18

**Authors:** Farah Zarka, Charles Veillette, Jean-Paul Makhzoum

**Affiliations:** ^1^Hôpital du Sacré-Cœur de Montreal, University of Montreal, Canada; ^2^Vasculitis Clinic, Department of Internal Medicine, Hôpital du Sacré-Coeur de Montreal, University of Montreal, Canada

## Abstract

Primary systemic vasculitides are rare diseases that may manifest similarly to more commonly encountered conditions. Depending on the size of the vessel affected (large vessel, medium vessel, or small vessel), different vasculitis mimics must be considered. Establishing the right diagnosis of a vasculitis mimic will prevent unnecessary immunosuppressive therapy.

## 1. Introduction

Vasculitides are rare heterogenous diseases that affect vessel walls as the main site of inflammation. Organs affected vary depending on the type and size of blood vessels involved [1]. Autoimmune vasculitis can be primary (idiopathic) or secondary to an underlying disease. Treatment usually consists of immunosuppressive therapy; treatment failure should raise concern for an alternative diagnosis before escalating therapy. There are many disorders, some of which are more common than vasculitides, which may mimic the clinical presentation and radiological or histologic features of vasculitides. It is important to recognize a vasculitis mimic to avoid unnecessary immunosuppressive therapy which may worsen the disease [2].

Vasculitides are classified according to the nomenclature system developed through the international Chapel Hill Consensus Conference (CHCC) and include large-vessel vasculitis (LVV), medium-vessel vasculitis (MVV), and small-vessel vasculitis (SVV) [3]. In this article, we will identify diseases that may mimic vasculitides according to the phenotype and size of the vessel affected. Common disorders in our daily clinical practice that can mimic vasculitides will be discussed in detail. CNS vasculitis mimics will not be discussed in this review.

## 2. Large-Vessel Vasculitis Mimics

Large-vessel vasculitis (LVV) is an inflammatory vasculopathy affecting large arteries; giant cell arteritis (GCA) and Takayasu's arteritis (TAK) are the two main documented variants, each with their own characteristic features. Associated aortitis can lead to aortic aneurysm formation, rupture, or dissection, while luminal narrowing of the aorta's main branches can result in various ischemic complications. Isolated aortitis is also a recognized entity classified as a single-organ vasculitis. It may be isolated, progress to GCA, TAK or be a manifestation of a systemic disease [3].

Giant cell arteritis predominantly affects the thoracic aorta as well as the carotid, vertebral, and axillary arteries. It occurs almost exclusively in individuals over the age of fifty. Patients typically present with constitutional symptoms, elevated inflammatory markers, headaches, scalp hyperesthesia, and jaw claudication. Approximately half of individuals diagnosed with GCA will present at some point with polymyalgia rheumatica. Associated arteritic ischemic optic neuropathy (ION) is one of the most feared complications [4]. Temporal artery biopsy has long been considered the diagnostic gold standard; however, colour Doppler ultrasound is becoming an alternative diagnostic tool.

Takayasu's arteritis primarily occurs in individuals under the age of forty. Patients initially may present nonspecific malaise, arthralgia, weight loss, and fever. With disease progression, symptoms of ischemic complications become more apparent. Subclavian artery narrowing is a common anomaly leading to limb claudication, diminished pulses, and inconsistent blood pressure measurements between the upper extremities. Vertebral arteritis can manifest as a range of neurological symptoms. Disease extension to the coronary ostia can lead to fatal myocardial infarction [5].

The diagnosis of LVV can be challenging as numerous pathologies present with similar clinical manifestations and radiological findings (Table 1). Early recognition is essential to avoid life-threatening vascular events and morbidity.

### 2.1. Infectious Aortitis

Tuberculous aortitis is a rare but morbid complication of active TB. Associated vascular anomalies are the result of hematogenous spread of *Mycobacterium tuberculosis* or vessel wall erosion by contiguous adenitis. Cases of true aneurysms and aortic narrowing have been reported, but pseudoaneurysms are by far the most common complication [6]. Untreated syphilis can lead to tertiary syphilis years after initially contracting the sexually transmitted disease. Tabes dorsalis, general paresis, gummas, and cardiovascular anomalies are the main consequences of late venereal disease. Syphilitic aortitis can cause ascending aortic aneurysms, aortic valve regurgitation, and coronary ostia narrowing. Vascular complications are mostly attributed to vasa vasorum disease. This vessel wall blood supply is typically found in the thoracic aorta adventitia layer, hence explaining abdominal aortic sparing [7].

### 2.2. IgG4-Related Disease

IgG4-related disease (IgG4-RD) is a systemic immune disorder with potential multiorgan dysfunction and failure. Lymphocytic infiltration with high IgG4 plasma cells, storiform fibrosis, and obliterative phlebitis are the three key histopathological findings [8]. Our understanding of this entity is continuously evolving, and only recently, classification criteria were proposed by an international panel of experts. Four distinctive phenotypes were described: pancreato-hepato-biliary disease, head and neck-limited disease, Mikulicz syndrome with systemic involvement, and retroperitoneal fibrosis and/or aortitis [9]. Vessel wall thickening and luminal dilation of the aorta and its main branches are the most commonly observed vascular anomalies [10].

### 2.3. Erdheim-Chester's Disease

Erdheim-Chester's disease is a non-Langerhans histiocytic disease with multisystemic manifestations. Its hallmark features include osteosclerotic bone lesions with foamy histiocytes on biopsy, retroperitoneal infiltration, periorbital disease, and cardiac pseudotumors. Associated vasculopathy can mimic inflammatory vasculitis. Reported anomalies include vascular ectasia, stenosis, and more commonly periarterial thickening throughout the aorta and its main branches [11].

### 2.4. Atherosclerosis

Degenerative aortic aneurysms tend to occur in older patients with no history of connective tissue disorders, systemic inflammatory diseases, or infectious aortitis. Numerous studies have linked aortic aneurysm formation to atherosclerosis. Yet, it remains uncertain if this is a direct association or a result of commonly known atherosclerotic disease risk factors [12].

Monocular vision loss can be caused by nonarteritic ischemic optic neuropathy. It is thought to be the result of vascular insufficiency rather than the inflammatory process seen in arteritic ION. Its underlying pathogenesis is most likely multifactorial. Atherosclerosis can cause a disruption in the optic disc's autoregulation, contributing to impaired blood flow. Known cardiovascular risk factors, particularly hypertension and diabetes, have been associated with nonarteritic ION [13]. Central retinal artery occlusion (CRAO) or branch retinal artery occlusion (BRAO) may also be arteritic ION mimickers. They are often the result of occlusive atherosclerotic plaques in the carotid or retinal artery or emboli from a distant source. CRAO or BRAO is considered the ocular counterpart of ischemic stroke with the same risk factors. Isolated visual symptoms are often a sign of an impending cerebral vascular event and should be managed with the same urgency [14].

### 2.5. Other

Some inflammatory vasculopathies are identified as variable vessel vasculitis (VVV) as they can affect small, medium, and/or large arteries [3]. SVV associated with Cogan's syndrome can lead to inner ear involvement and ocular disease in the form of uveitis, scleritis, episcleritis, or conjunctivitis [3]. Its large-vessel vasculopathy can cause aortic aneurysms and left-sided heart valvulitis [3, 15]. Behçet's syndrome is also a VVV affecting both arteries and veins. Some of its cardinal features include genital and oral aphthous ulcers, erythema nodosum, pseudofolliculitis, uveitis, retinal vasculitis, and pathergy. Arterial involvement is a significant cause of morbidity and mortality in this population. Typical vascular complications include aortic, pulmonary, and peripheral artery aneurysms and inflammatory arterial and venous thromboses [3, 16].

Vasculitis associated with systemic diseases refers to an inflammatory vasculopathy caused by another systemic disorder [3]. A few conditions tend to particularly affect large vessels. Rheumatoid aortitis is a rare complication of long-standing untreated seropositive disease [17]. Relapsing polychondritis (RPC) is known for its multisystem inflammation of cartilaginous structures. Its most common macrovascular complications include aortic aneurysms and regurgitation [18]. Patients with spondyloarthropathies can develop cardiovascular complications in advanced stages of the disease; associated aortitis typically affects the ascending aorta as well as the aortic root, causing annulus dilation and valvular regurgitation [19].

### 2.6. Genetic Disorders

Genetic conditions can mimic large-vessel vasculitis when vascular complications arise. However, they lack systemic inflammation, a cardinal feature often associated with LVV (Table 2).

## 3. Medium-Vessel Vasculitis Mimics

The two medium-vessel vasculitides according to the CHCC [3] are polyarteritis nodosa (PAN) and Kawasaki disease (KD).

PAN is an ANCA-negative necrotizing vasculitis that is most commonly systemic, although it may be limited to the skin (4–5% of cases) [24, 25]. Its etiology is unknown although there is a strong association with viral infections (especially hepatitis B). It has also been observed in patients with DADA2 (deficiency of adenosine deaminase 2). Cutaneous manifestations include erythematous subcutaneous nodules, necrotic ulcers, and livedo reticularis. Systemic manifestations include constitutional symptoms, myalgias, arthralgia, abdominal pain, kidney infarcts, orchitis, and peripheral neuropathy (mononeuritis multiplex or polyneuropathy). Microaneurysms are a hallmark of the disease and can be detected by angiography or biopsies. Diagnosis relies on the combination of clinical findings with typical imaging and biopsy results.

Kawasaki disease, also called mucocutaneous lymph node syndrome, is a necrotizing vasculitis typically seen in infants and children. It can sometimes be seen in adults and presents with lymphadenopathy, hepatitis, arthralgia, constitutional symptoms, and coronary artery involvement. A long-term follow-up with a cardiologist is recommended to assess regularly for the onset of complications such as persistent coronary aneurysms and accelerated atherosclerosis [26, 27]. Mimics of MVV are presented in Table 3.

### 3.1. Fibromuscular Dysplasia

Fibromuscular dysplasia (FMD) is an idiopathic segmental nonatherosclerotic, noninflammatory arteriopathy that predominantly affects women [28, 29]. Classification is based on angiographic appearance: either focal or multifocal (string of beads) [30]. Aneurysms, dissections, and tortuosity can be found in all arteries, but renal, internal carotid, and intracerebral arteries are mostly involved [31]. Two or more vascular beds are usually affected. Clinical manifestations include hypertension, headache, tinnitus, dizziness, and vascular bruits on auscultation [31]. The most severe complications are acute kidney injury, renal infarction, transient ischemic attack, stroke, myocardial infarction, and mesenteric ischemia. Diagnosis is usually achieved in the presence of clinical manifestations and compatible vascular imaging [28, 30, 32, 33].

### 3.2. Segmental Arterial Mediolysis

Segmental arterial mediolysis (SAM) is an idiopathic disease that causes degeneration and lysis of the myocytes' vacuoles in the medial layer of arteries [34]. A single vascular bed is usually affected in a person; it affects mainly the visceral (especially the celiac artery), retroperitoneal, and renal beds [35, 36]. Intracranial and coronary vessels can also be involved [34, 37]. Unlike PAN, there is an absence of systemic inflammation. SAM affects men and women almost equally, as opposed to FMD. Numerous cases are discovered incidentally on imaging; however, hemorrhages from dissection or ruptured aneurysms are often the manifestation of SAM [35, 38]. This feature allows distinguishing SAM from FMD, which is mainly an occlusive disorder. Angiographic findings include arterial dilations, single or multiple aneurysms, intramural hematoma, arterial stenosis, or, rarely, arterial occlusions [34, 38].

### 3.3. Infectious Endocarditis and Mycotic Aneurysms

Infectious endocarditis (IE) and mycotic aneurysms (MA) can mimic primary vasculitis and confound even experienced clinicians due to the similar multisystemic and progressive symptoms. Constitutional symptoms are common, and positive ANCA can be detected in around 25% of the patients with infectious endocarditis which can be misleading [39–42]. ANCA-positive endocarditis is more prevalent in men with a median age of onset of 53.5 years. It is believed that microorganisms induce an immune response with antibody synthesis causing ANCA positivity [42]; there is no explanation yet as to why only a fraction of endocarditis exhibits this feature. Some pathogens seem to be more likely associated with ANCA-positive IE, especially Bartonella [43]. Echocardiographic findings do not differ between positive and negative ANCA IE, but there seems to be more renal involvement with a positive ANCA [40, 42]. A rare complication of IE is mycotic aneurysms that occur because of septic embolization to the arterial vasa vasorum with blood supply interruption and subsequent wall weakness. A distinctive feature of mycotic aneurysms is that they affect predominantly the arteries' bifurcations [34, 37, 44, 45]. The diagnosis of IE relies on Duke's modified criteria [46, 47], and treatment is based on appropriated antibiotics and sometimes requires surgery to remove vegetation or to repair valve defects [45, 47].

## 4. Small-Vessel Vasculitis Mimics

SVV is defined as vasculitis that typically affects small vessels such as arterioles, venules, and capillaries [3, 48]. Nevertheless, SVV may less frequently affect medium or large vessels, as in LVV and MVV. Every organ system can be affected in SVV including eyes, ENT, lungs, kidneys, skin, joints, and nervous system [48]. Constitutional symptoms such as fatigue, low-grade hyperthermia, and weight loss can be present as in other infectious, neoplastic, or inflammatory conditions [49].

SVV can be further divided into ANCA-Associated SVV (AAV) and Immune Complex SVV (IC-SVV). AAV includes Granulomatosis with Polyangiitis (GPA, formerly Wegener's granulomatosis), Microscopic Polyangiitis (MPA), and Eosinophilic Granulomatosis with Polyangiitis (EGPA, formerly Churg-Strauss). IC-SVV includes cryoglobulinemic vasculitis, IgA Vasculitis (IgAV, formerly Henoch-Schönlein), antiglomerular basement membrane (anti-GBM) disease, and urticarial vasculitis [3].

Because clinical presentation of SVV is heterogenous, we will discuss SVV mimics according to clinical phenotypes commonly encountered in the clinical setting: isolated skin vasculitis, pulmonary-renal syndrome, asthma with eosinophilia, and midline destructive lesions (Table 4).

### 4.1. Isolated Skin Vasculitis Mimics

Cutaneous manifestations of vasculitis include purpura, urticarial lesions, nodules, ulcers, livedo reticularis, and livedo racemosa [50]. Because the skin lesion is often nonspecific, a skin biopsy is usually required to prove or exclude vasculitis. Nevertheless, clinicopathological correlation is a key to establish the correct diagnosis since the presence of histologically proven leukocytoclastic vasculitis can be found in nonvasculitic conditions such as drug reactions, insect bite reactions, viral infections, and neutrophilic dermatosis [51].

Purpura can be observed in stasis dermatitis due to venous incompetence which increases hydrostatic pressure and causes capillary damage with extravasation of red blood cells [52]. This can be exacerbated by antiplatelet or anticoagulation therapy. Similarly, cellulitis can be accompanied with purpura due to soft-tissue inflammation and edema [53]. Thrombotic disorders should be considered in the setting of purpura, such as antiphospholipid disease, purpura fulminans (in the setting of sepsis and disseminated intravascular coagulopathy), and thrombotic thrombocytopenic purpura [54]. Embolic diseases must also be considered in the setting of purpura and include cholesterol emboli, infectious endocarditis septic emboli, and embolic atrial myxoma. A complete blood count with differential and platelets, coagulation tests (INR, aPTT, and fibrinogen), liver and renal function tests, and urinalysis are useful in the differential diagnosis. Blood cultures and echocardiography should be obtained when an infection is suspected [55].

Hives can be observed in urticarial vasculitis but are most commonly found in the setting of allergic or nonallergic urticaria. Urticaria can be acute (duration of less than 6 weeks) or chronic. Common causes of urticaria include infections and allergic reactions to medications, foods, plants, or insect bites [56]. Viral exanthems may also cause urticarial lesions, particularly in children. Serious systemic conditions may also present with urticaria such as systemic mastocytosis and autoinflammatory diseases.

The presence of skin nodules must raise suspicion for infectious and noninfectious granulomatous diseases, panniculitis (such as erythema nodosum), and neoplastic diseases [57].

Chronic ulcers may raise the suspicion for vasculitis. However, venous hypertension and peripheral arterial disease are more frequently encountered. Venous ulcers typically occur along the medial or lateral distal leg above the ankle. Arterial ulcers have more round and regular shapes and occur more distally, typically on the toes or in the lateral foot, but can occur anywhere on the leg [58]. Pyoderma gangrenosum (PG) is a rare inflammatory condition that presents with ulcers, characterized by a violaceous contour and an undermined quality. PG occurs more frequently on the legs but can appear on the location of surgery or trauma. Most cases of PG are idiopathic, although approximately 50% of cases are associated with other disorders, especially inflammatory bowel diseases [59].

The presence of livedo reticularis and livedo racemosa can occur in the context of AAV and cutaneous polyarteritis nodosa. Nevertheless, many conditions that may be life-threatening need to be considered (Table 4) [60].

### 4.2. Pulmonary-Renal Syndrome Mimics

The pulmonary-renal syndrome (PRS) involves the combination of diffuse alveolar hemorrhage (DAH) with rapid progressive glomerulonephritis (RPGN) and usually occurs in the setting of AAV or anti-GBM disease. Clinical presentation varies as symptoms do not always occur simultaneously. Pulmonary symptoms include cough, dyspnea, chest pain, and hemoptysis. Microscopic hematuria with renal function loss and nephritic syndrome usually progresses rapidly [61].

Severe infections with multiorgan failure can mimic PRS. Acute kidney injury (AKI) due to sepsis combined with fluid overload (sometimes exacerbated by septic cardiomyopathy) may cause pulmonary edema and DAH. Infectious endocarditis can cause septic emboli to the kidneys (AKI with hematuria) and lungs, thus mimicking PRS. Other infections can directly affect lungs and kidneys such as tuberculosis, Mycoplasma, Legionella, and Cytomegalovirus.

Thrombotic disorders such as antiphospholipid syndrome may present with pulmonary embolism and hemoptysis along with AKI due to glomerular capillary, renal artery, or vein thrombosis. Similarly, a thrombotic thrombocytopenic purpura can progress rapidly, mimicking PRS. Renal vein thrombosis (with AKI) in the setting of a nephrotic syndrome, with fluid overload, pulmonary edema, and hemoptysis, may clinically present like a PRS [62].

Other nonvasculitic autoimmune diseases can involve lungs and kidneys. As an example, systemic lupus erythematosus (SLE) may present with lupus nephritis and interstitial lung disease which could be radiologically indistinguishable from DAH.

### 4.3. Asthma and Eosinophilia (EGPA Mimics)

The diagnosis of EGPA is usually straightforward if the patient presents with chronic sinusitis, asthma, and eosinophilia in association with systemic findings of vasculitis (skin, lungs, kidneys, neuropathy, etc.) [63]. However, vasculitis is not always present and alternative diagnoses must be considered.

Hypereosinophilic asthma represents a more severe phenotype of asthma and occurs in up to 10% of patients with adult-onset asthma. Although tissue and circulating levels of eosinophils are high, these patients rarely have a history of atopy [64]. The entire respiratory tract might be involved, from sinuses to distal airways. Affected patients are often refractory to conventional inhaled therapy and require systemic glucocorticoids early in the disease course; thus, referral to an asthma expert is imperative. Eosinophil levels in sputum can help to individualize treatment which includes novel therapies such as anti-IL-5 agents [65].

Allergic bronchopulmonary aspergillosis (ABPA) is a complex hypersensitivity reaction caused by colonization of the airway with Aspergillus fumigatus. It occurs in patients with asthma (up to 5%) and in patients with cystic fibrosis (up to 10%) [66]. Bronchial mucoid impactions occur, and areas of eosinophilic pneumonia are sometimes found. Patients present with recurrent asthma exacerbations, constitutional symptoms, and expectoration of brownish mucus plugs. Laboratory findings may include eosinophilia, elevated total IgE, and elevated Aspergillus-specific IgE on immunoassays. An Aspergillus skin test should be used in patients with suspected ABPA [67]. Chest radiograph and computed tomography of the lungs may reveal pulmonary infiltrates in a pattern consistent with ABPA [68].

Aspirin-exacerbated respiratory disease (AERD) is a reaction to acetylsalicylic acid and other NSAIDs (COX-1 inhibitors). It refers to the combination of chronic rhinitis with nasal polyposis and asthma in patients taking aspirin [69]. These manifestations tend to develop successively over a period of years although rapid progression is possible. Eosinophilia is present in approximately 50% of cases and may correlate with AERD severity. No other organ system involvement is present in AERD [70].

Idiopathic eosinophilic pneumonia is a rare condition. Patients usually present with an acute illness of less than 4 weeks (fever, dyspnea, and unproductive cough), hypoxemic respiratory failure, diffuse pulmonary opacities on chest radiograph, and bronchoalveolar lavage showing eosinophilia > 25%. Other causes of eosinophilic pneumonia must be excluded (drugs, infections, EGPA, and atopic disease) [71].

Hypereosinophilic syndrome (HES) implies the presence of hypereosinophilia with eosinophilic organ damage. HES can be primary (hematological clonal disease), secondary (in the setting of parasitic infections or certain tumors), or idiopathic. Elevated vitamin B12, anemia, thrombocytopenia, and hepatosplenomegaly may hint towards HES, but this is not mandatory. A hematology evaluation with advanced cytogenetic and molecular testing is often required to prove the diagnosis [72, 73].

### 4.4. Midline Destructive Lesions

Cocaine-induced midline destructive lesions (CIMDL) are due to a direct vasoconstrictor effect of cocaine and are sometimes difficult to differentiate from GPA's localized sinonasal involvement. CIMDL involves destruction of the osseocartilaginous structures of the nose, sinuses, and palate [74]. Trimarchi et al. compared features of 18 consecutive patients with CIMDL with those of 21 patients with GPA [75]. CIMDL tends to be more severe, and no other organ involvement or systemic inflammation is present as opposed to GPA. Histologic features on biopsy are rarely useful to differentiate both entities as leukocytoclastic vasculitis, and fibrinoid necrosis may be present in CIMDL. In patients with CIMDL with positive p-ANCA, none reacted to MPO, but four reacted to PR3, three reacted to human neutrophil elastase (HNE), and two patients had double positivity to PR3 and HNE. In contrast, patients with GPA usually have concurrent reactivity (p-ANCA/MPO-ANCA and c-ANCA/PR3-ANCA).

## 5. Conclusion

Recognizing conditions that mimic clinical, laboratory, radiological, and histologic features of primary systemic vasculitides is essential to avoid unnecessary immunosuppression and to treat the correct disease. A multidisciplinary approach is often required to appropriately diagnose vasculitis mimics.

## Figures and Tables

**Table 1:** Conditions that mimic LVV.

Infectious
Tuberculous aortitis
Vascular anomalies include true aneurysm formation, aortic narrowing, and pseudoaneurysm caused by hematogenous dissemination and/or vessel wall erosion
Syphilitic aortitis
Tertiary syphilis can lead to ascending aortic aneurysm, aortic valve regurgitation, and coronary ostia narrowing
IgG4-related disease
IgG4 plasma cell infiltration causes vessel wall thickening and luminal dilation of the aorta and its main branches
Erdheim-Chester's disease
Commonly associated vascular anomalies include vascular ectasia, stenoses, and periarterial thickening of the aorta and its main branches
Atherosclerosis
Degenerative aortic aneurysm
Central retinal artery occlusion or branch retinal artery occlusion
Acute monocular vision loss caused by atheromatous plaques or distal emboli mimicking arteritic ischemic optic neuropathy
Nonarteritic ischemic optic neuropathy
Monocular vision loss triggered by vascular insufficiency and disruption of the optic disc's autoregulation
Variable vessel vasculitides
Cogan's syndrome
Associated large-vessel vasculopathy can lead to aortic aneurysms and left-sided heart valvulitis
Behçet's syndrome
Vascular complications include aortic, pulmonary, and peripheral artery aneurysms, arterial and venous thromboses, or thromboangiitis
Vasculitis associated with systemic disease
Rheumatoid aortitis
Aortitis caused by long-standing untreated seropositive disease
Relapsing polychondritis
Aortitis leading to aortic aneurysms at risk of rupture and/or dissection
Seronegative arthritis
Aortitis associated with ankylosing spondylitis and peripheral spondyloarthropathies can lead to ascending aortic aneurysm, aortic root annulus dilation, and valvular regurgitation
Other conditions
Fibromuscular dysplasia
Often involves renal arteries but may also affect large arteries including carotid, vertebral, and intracranial arteries
Segmental arterial mediolysis
Most commonly involves medium-sized abdominal visceral arteries but may rarely affect all vessels including carotid arteries with a risk of cerebral infarction

**Table 2:** Overview of genetic disorders mimicking LVV.

	Marfan syndrome (MFS) [20]	Loeys-Dietz syndrome (LDS) [21]	Ehlers-Danlos syndrome (EDS) [22]	Neurofibromatosis type 1 (NF-1) [23]
Definition	Autosomal dominant heritable connective tissue disorder caused by a fibrillin 1 (FBN1) gene mutation	Autosomal dominant heritable connective tissue disorder caused by a mutation in the transforming growth factor *β* receptor 1 or 2 (TGFBR1/TGFBR2) genes	Autosomal dominant connective tissue disorder caused by mutations in the genes encoding type I or III procollagen (COL1A1/COL3A1). Thirteen subtypes are recognized with subtype four known as vascular EDs (vEDS)	Autosomal dominant genetic disorder caused by a mutation in the neurofibromin 1 (NF1) tumor-suppressor gene

Main clinical features	(i) Aortic root dilation (ii) Aortic root dissection (iii) Ectopia lentis (iv) Spontaneous pneumothorax (v) Mitral valve prolapse (vi) Musculoskeletal anomalies (hindfoot deformity, pectus carinatum or excavatum, scoliosis, and arachnodactyly)	(i) Hypertelorism (ii) Bifid uvula or cleft palate (iii) Arterial tortuosity and aneurysms that can involve the aorta, head, neck, and abdominal arteries	(i) Dissection or spontaneous arterial rupture occurring in young patients (ii) Spontaneous sigmoid colon perforation (iii) Uterine rupture during pregnancy (iv) Carotid-cavernous sinus fistula formation (v) Other minor criteria include skin, muscle, bone, and joint anomalies	(i) Café-au-lait spots (ii) Axillary freckling (iii) Optic pathway gliomas (iv) CNS tumors (v) Lisch nodules (vi) Neurofibromas (vii) Learning disabilities (viii) Aneurysms (ix) Arterial stenoses (x) Aortic coarctation (xi) Arteriovenous malformations

**Table 3:** Comparison of conditions that mimic MVV.

	SAM	FMD	PAN	KD	IE/MA
Age of presentation	40–60	20–30	40–60	0–5	Any
Sex predisposition (F : M)	Equal	3 : 1	Equal	1 : 1.5	Equal
Most frequent anatomical sites	Visceral mesenteric arteries	Renal arteries	Intra-abdominal arteries	Coronary arteries	Any
Radiological features	“String of beads” appearance and dissections with alternating stenosis and aneurysms, thrombosis	Classic “string of beads” appearance with alternating stenosis and aneurysms, rarely dissections	Characteristic microaneurysms predominantly at vessel branch points	Coronary aneurysms in 15–20% of cases	Pseudoaneurysms at branch points
Histologic features	Media predominantly involved with alternating stenoses and aneurysms	All vessel layers may be affected Pattern depends on histologic subtype	Focal areas of necrotizing panarteritis	Perivasculitis and vasculitis of microvessels with vessel wall edema	Infectious elements present

**Table 4:** Conditions that mimic SVV.

Isolated skin vasculitis mimics
Purpuric lesions
Drug eruptions, erythema multiforme, arthropod bites, infectious emboli (endocarditis), pigmented purpura, cellulitis, viral exanthems, hemorrhagic lesions, scurvy, antiphospholipid syndrome, heparin-induced thrombocytopenia, warfarin necrosis, thrombotic thrombocytopenic purpura, paroxysmal nocturnal hemoglobinuria, amyloidosis
Urticarial lesions
Urticaria, arthropod bites, serum sickness-like eruption, autoinflammatory diseases, Sweet's syndrome, viral exanthems, Schnitzler's syndrome
Nodular lesions
Panniculitis, granulomatous diseases, cutaneous neoplasm, superficial phlebitis
Ulcers
Venous hypertension, peripheral artery disease, pyoderma gangrenosum, calciphylaxis, hypercoagulable diseases, livedoid vasculopathy, chronic infections, hemoglobinopathies
Livedo reticularis
Paraproteinemia, livedoid vasculopathy, purpura fulminans, hypercoagulable diseases, thrombotic microangiopathies
Pulmonary-renal syndrome mimics
Infectious disease mimics
Severe bacterial pneumonia, postinfectious glomerulonephritis, infective endocarditis, sepsis with multiorgan failure (ARDS and AKI)
Cardiac mimics
Pulmonary edema with cardiorenal AKI
Renal mimics
Nephrotic syndrome with pulmonary emboli, microangiopathic diseases
Connective tissue diseases
Systemic lupus erythematosus, mixed connective tissue disease, systemic sclerosis with scleroderma renal crisis
Antiphospholipid syndrome
Asthma and eosinophilia (EGPA mimics)
Infectious diseases
Toxocariasis, HIV, Aspergillus spp.
Hematological diseases
Hypereosinophilic syndrome, eosinophilic leukemia, systemic mastocytosis, chronic myeloid leukemia
Paraneoplastic syndrome
Lung cancer, cervical cancer, Hodgkin's lymphoma, non-Hodgkin's T-cell lymphoma
Pulmonary diseases
Hypereosinophilic asthma, allergic bronchopulmonary aspergillosis, aspirin-exacerbated respiratory disease, idiopathic eosinophilic pneumonia
Midline destructive lesions (ENT limited GPA mimics)
Cocaine-induced midline destructive lesions
Necrotizing sinonasal infections
Usual ENT bacteria, mycobacteria, syphilis, fungal, parasitic
Neoplastic
NK/T-cell lymphoma
Inflammatory
Sarcoidosis, IgG4-related disease
Idiopathic midline destructive disease

## Additional Points

*Take-Home Messages*. (1) Mimics of vasculitides must be excluded during the investigation of a possible primary vasculitis. (2) Atypical features or lack of response to treatment must raise suspicion of an alternative diagnosis. (3) The differential diagnosis of large-vessel vasculitis most commonly includes atherosclerosis, infectious aortitis, and genetic disorders. (4) Vascular imaging and inflammatory markers are often useful in the evaluation of a medium-vessel vasculitis mimic. (5) Clinical presentation of small-vessel vasculitides is heterogenous, and multidisciplinary collaboration is required to confirm the correct diagnosis.
